# Adolescent and Youth Experiences With Contraceptive Self-Injection in Uganda: Results From the Uganda Self-Injection Best Practices Project

**DOI:** 10.1016/j.jadohealth.2022.08.010

**Published:** 2023-01

**Authors:** Caitlin Corneliess, Jane Cover, Andrew Secor, Allen Namagembe, Fiona Walugembe

**Affiliations:** a PATH, Sexual and Reproductive Health Team, Seattle, Washington; b PATH, Sexual and Reproductive Health Team, Kampala, Uganda

**Keywords:** Self-care, Self-injection, Sexual and reproductive health and rights, Family planning, Contraception, Adolescents, DMPA-SC

## Abstract

**Purpose:**

We used qualitative and quantitative data to evaluate the differing experiences of adolescents and adult women in the contraceptive self-injection program in primary care settings in Uganda. From these results, we assessed barriers to adolescent DMPA-SC self-injection access and continuation and provide recommendations to address them.

**Methods:**

The Self-Injection Best Practices (2017–2019) project in four districts trained clinic-based providers and Village Health Teams to provide self-injection training in clinics, community settings, and small group meetings for adolescent girls and young women. More than 12,000 women of reproductive age received self-injection services through the program, including 2,215 under 20 years. Structured surveys (n = 1,060) and in-depth interviews (n = 36) were conducted with randomly selected adolescent participants between July and November 2018. Mixed-effects logistic regression was used to assess quantitative differences in outcomes of interest between age groups.

**Results:**

The study found no significant difference in self-injection proficiency or continuation between adolescents and adult women; 86.1% of adolescents self-injected independently when due for reinjection. Adolescents were significantly less likely than adults to report first hearing about self-injection from a community health worker. More adolescents expressed concern over discovery when seeking contraception at a clinic and fear of their DMPA-SC units being discovered at home. Adolescents were significantly less likely than adult women to mention convenience as a rationale for self-injecting, and more likely to mention wanting to learn a new skill and/or that friends recommended self-injection.

**Discussion:**

Self-injection is a promising method of contraception for adolescents in Uganda, given comparable proficiency and continuation relative to adult women. Policies and programs should ensure rights-based access to a range of methods, including self-injection for this age group.

**Implications and Contribution**

Uganda was the first country in sub-Saharan Africa to offer contraceptive self-injection outside of a research setting. The results presented provide an understanding of the experiences of adolescent girls and young women (aged 15–24 years) with self-injection in a primary care setting and how they differ from those of adult women. In addition, the results provide insights that can assist in the expansion of contraceptive choice and access for adolescents in a context where the adolescent pregnancy rate is high.

Uganda's policy environment is receptive to adolescent sexual and reproductive health (SRH); nonetheless, implementing programs to improve access to contraceptive services for adolescents remains challenging as evidenced by the relatively high childbearing rate. Among adolescents aged 15–19 years, one-quarter have begun childbearing [1].

Innovations are providing new opportunities for adolescents to exercise self-efficacy and autonomy in obtaining health services [2]. In 2019, the World Health Organization released guidelines on self-care for SRH and rights, including evidence-based recommendations that can be used by people globally to help advance health equity—supported by high-quality, rights-based programs and services [3]. The guidance strongly recommends that self-administered injectable contraceptives be made available for individuals of reproductive age.

Subcutaneous depot medroxyprogesterone acetate (DMPA-SC; Pfizer Inc.’s Sayana® Press) is a 3-month, progestin-only injectable contraceptive packaged in the BD Uniject™ injection system, a small, prefilled, autodisable device designed to be suitable for self-injection (SI). Since 2015, DMPA-SC SI has been approved by regulatory authorities in more than 50 countries, including at least 30 low- and middle-income countries [4]. DMPA-SC was approved for SI by the Uganda National Drug Authority in 2017 and subsequently included in the national scale-up plan for SI. Uganda was the first country in sub-Saharan Africa to offer contraceptive SI outside of a research setting, with the key objective of understanding how women would learn to self-inject in a primary care setting.

DMPA-SC SI can improve adolescent access to a highly accepted method of contraception in the context of a broader contraceptive method mix [5]. Eliminating the need to obtain injectable contraception from a health worker could reduce barriers related to provider attitudes and misconceptions, lack of privacy and youth-friendly services at health facilities, travel costs and distance, and the shame or fear of discovery experienced by some adolescents when obtaining services [[6], [7], [8]]. In addition, storing multiple cycles of injectable contraception at home may improve contraceptive continuation rates, including among adolescents. Adolescents are particularly receptive to new technology [9]; and when asked, adolescents in Gulu District, Uganda, were receptive to the idea of SI [10]. In the first districts in Uganda where SI was rolled out in the public sector, 56% of clients were aged <25 years [11].

Although a growing number of studies have explored self-administration of injectable contraceptives in adult women [[10], [11], [12]], fewer have explored acceptability of DMPA-SC SI among adolescents in low-resource settings [12].

## Methods

### Study design and sample

In partnership with the Ugandan Ministry of Health, PATH developed, implemented, and evaluated a pilot program to generate evidence and guidance to design and implement SI programs in low-resource settings. The purpose of the evaluation was to assess the effectiveness and feasibility of the SI program, focusing on injection competency as the key measure of effectiveness (e.g., a successful SI client and by extension, a successful program approach). In addition, we wished to better understand the nature and content of client training, satisfaction with training, the nature of posttraining follow-up, and experiences with independent SI. Clinic-based providers and community health workers (called Village Health Teams or VHTs) in Gulu, Mayuge, Mubende, and Oyam Districts were trained to provide SI training in clinics; community settings; and in Mubende, Safe Spaces (Public areas such as schools and community centers, where girls and young women meet with female mentors and providers to receive interventions and services as part of, human immunodeficiency virus prevention programming.). More than 12,000 women, including 2,200 adolescents aged <20 years, received training in SI through the Self-Injection Best Practices project, and 56% of these women went on to become SI clients, either self-injecting or taking units home for future SI. Presented here are the results of structured surveys and in-depth interviews (IDIs) conducted between July and November 2018.

Evaluation participants were 1,060 adolescents aged 15–19 years (n = 208), young adults aged 20–24 years (n = 302), and adult women aged 25–55 years (n = 550) receiving SI training from a provider (clinic based or VHT) affiliated with a study site. Thirty-six adolescents also completed IDIs. To be eligible to participate in the evaluation, adolescents aged <18 years had to be emancipated (defined in Uganda as individuals below the age of majority who are pregnant, married, have a child, or are financially self-sufficient), and all participants were trained at a study site (or in the local community) and either self-injected posttraining or took units home for future SI. Study participants were randomly selected from among those entered into a SI training register and had agreed to be contacted for an interview.

### Study procedure and data collection

Participants were followed up after the date of their scheduled reinjection at home (3–4 months after their initial SI training). Research assistants conducted face-to-face interviews with participants in English or the primary language spoken in the area, following a structured survey, and entered data electronically using Open Data Kit. The instrument focused on motivations to try SI; training experience; and experience with independent SI posttraining, including challenges, whether they self-injected independently, and reasons for discontinuing. Participants were also asked to demonstrate their injection technique (on a model), which was evaluated by study staff using an observation checklist.

Proficiency was defined as satisfactorily completing four critical injection steps: (1) mixing the liquid; (2) breaking the seal between the reservoir and needle; (3) pinching the skin to form a “tent”; and (4) squeezing the reservoir slowly to inject. Clients were not given instructions during their demonstration but were encouraged to use the job aid.

A subsample of adolescent participants was selected for qualitative IDIs. Selection for participation in the IDIs was purposive, preferencing individuals who were more forthcoming with opinions during the survey interview; favorability toward the program did not influence participant selection. The qualitative lines of inquiry were focused on the same topics as the survey in more depth—awareness of SI, motivation to use SI, concerns around confidentiality, training experience and support needs, experience with reinjection, challenges with injecting, reasons for discontinuing, and storage and disposal practices. Interviews were audio recorded and transcribed into English.

### Data analysis

Quantitative data were analyzed using Stata IC/14.2 and R (v4.0.0) software. Chi-square and two-sided Student's *t*-tests were used to evaluate differences between groups. Mixed-effects logistic regression models were used for all other analyses, with nested random intercepts for the district and location of service (clinic) to account for within-cluster correlation. All statistical tests used an alpha level of 0.05 to determine statistical significance. Qualitative interviews were analyzed using Atlas.ti (v8) software. We followed an iterative process to develop a coding scheme based on emerging themes. The scheme was reviewed by a second coder, and discrepancies were resolved through consensus.

### Ethical considerations

All participants provided voluntary, written informed consent to participate in the evaluation. The study was approved by the Mulago Hospital Research and Ethics Committee and the Uganda National Council for Science and Technology.

## Results

### Participant overview

Adolescent participants differed from women age ≥25 years in a number of ways (Table 1). More adolescents were single (37.5% vs. 8.5%), living with a parent or grandparent (33.2% vs. 6.7%), of lower parity (21.2% without children vs. 0.4%), and had reached at least secondary school (28.9% vs. 22.9%). More were first-time users of a modern contraceptive method when they opted for SI (56.7% vs. 10.2%). Adolescents were significantly more likely to have last used condoms or pills than older women. Predictably, given stigma surrounding adolescent, particularly premarital, sexual activity, more adolescents than women aged ≥25 years (25.5% vs. 5.3%) expressed a high level of concern over possible discovery when seeking contraceptive methods at a clinic. Adolescents were less likely to have a partner who was aware of their use of self-injectable contraception; for those whose partner was aware, they were less likely to have their support. Similarly, fewer adolescents had partners very supportive of contraceptive use more generally.

**Table 1:** Characteristics of participants (quantitative survey)

	15–19 (n = 208)		20–24 (n = 302)		25+ (n = 550)		*p* value
	n/mean	%/SD	n/mean	%/SD	n/mean	%/SD	
Age (mean years, SD)	18.1	0.9	22.0	1.5	30.8	4.9	
District							
Gulu	34	16.3	103	34.1	184	33.5	
Mayuge	46	22.1	93	30.8	182	33.1	
Mubende	94	45.2	4	1.3	0	0	
Oyam	34	16.3	102	33.8	184	33.5	
Married or cohabitating with partner	130	62.5	260	86.1	503	91.5	<.001
Living with parent or grandparents	67	33.2	50	16.6	37	6.7	<.001
Living with friends, other family, or alone	24	11.5	13	4.3	55	10.0	.005
Parity (mean, SD)	1.1	0.4	2.0	1.0	4.2	1.8	<.001
Parity							<.001
0 children	44	21.2	7	2.3	2	0.4	
1 child	144	69.2	107	35.4	15	2.7	
2+ children	20	9.6	188	62.3	533	96.9	
Education (mean years, SD)	6.8	2.1	6.6	2.7	6.0	3.2	<.001
Education							.006
None	2	1	10	3.3	35	6.4	
Primary	146	70.2	215	71.2	389	70.7	
Secondary or higher	60	28.9	77	25.5	126	22.9	
First-time user any FP method	118	56.7	95	31.5	56	10.2	<.001
Last FP method used, if any							
Condom	14	15.4	11	5.4	16	3.2	<.001
Pill	10	11.0	16	7.8	21	4.3	.019
LARC (IUD or implant)	8	8.8	17	8.3	57	11.6	.378
DMPA-IM	42	46.2	112	54.6	291	59.0	.063
DMPA-SC	14	15.4	46	22.4	94	19.1	.338
Other	3	3.3	3	1.5	14	2.8	.508
Privacy concerns obtaining FP from health facility							<.001
Not at all concerned	131	63.0	224	80.8	465	84.5	
A little concerned	24	11.5	45	14.9	56	10.2	
Very concerned	53	25.5	13	4.3	29	5.3	
Partner aware of self-injection use (yes)	146	70.2	241	79.8	416	75.6	.045
Partner supportive of self-injection, if aware of use (yes)	101	71.6	225	90.4	371	84.5	<.001
Partner supportive of FP use (yes, very)	127	66.5	227	79.4	388	73.5	.007

DMPA-IM = intramuscular depot medroxyprogesterone acetate; DMPA-SC = subcutaneous depot medroxyprogesterone acetate; FP = family planning; IUD = intrauterine device; LARC = long-acting reversible contraception; SD = standard deviation.

### Privacy, where heard about SI, and motivation for SI

The IDIs revealed considerable fear of discovery of DMPA-SC units and used needles stored at home. Adolescents mentioned concerns about family and community members learning they were self-injecting.

*I am so worried because it can cause me problems even if it is not the parent who has found it but that person who sees it can tell my parent. That’s the worry I have.* [15-year-old single female]

*They would say, “See that girl over there self injecting, she is a prostitute.”* [15-year-old single female]

*Only my mother knows that am using Sayana Press. It would be a big problem if other people like my father and sisters found out… They start talking about you, that you are on family planning…things like that!* [17-year-old single female]

*For me personally and my husband, we are the only ones who know…and do not want others to know, or to hear it from others, that we are on family planning. If I am careless with my Sayana units and someone finds them, they may say, “eeeh all along you have been on family planning.” So [they] may not treat us well if others get to know.* [18-year-old married female]

*I kept them at my friend’s place, because here at home I am not allowed family planning; I am on it stealthily…. My friend too uses family planning and her husband is aware…* [19-year-old single female]

Adolescents also differed from older participants in terms of how they first learned about SI (Table 2). The odds of hearing about SI from a VHT were significantly lower for adolescents (27% lower) and young women (16% lower) compared with adult women. Conversely, the odds of adolescents hearing about SI from friends or family were twice as high as for adult women.

*I first heard of it from a friend. I had decided to go to a clinic and buy an injectable, then a friend told me that she comes to a government facility, where they give free injections for people to self inject. She told me it is good and it has no problem. So I said let me go to the facility they give me the injection I self-inject.* [18-year-old married female]

**Table 2:** How women first hear about self-injection and motivation for learning to self-inject

		Age group	Odds ratio	CI (95%)	*p* value
Where first heard about self-injection	Village health team	25+	Ref	—	—
		15–19	0.73	0.73–0.73	<.001
		20–24	0.84	0.84–0.85	<.001
	Friend or family member	25+	Ref	—	—
		15–19	2.1	1.34–3.29	.001
		20–24	1.1	0.73–1.65	.641
	Educational talk at a health center	25+	Ref	—	—
		15–19	0.81	0.49–1.33	.397
		20–24	1.10	0.78–1.57	.578
	During family planning counseling at a health center	25+	Ref	—	—
		15–19	0.68	0.42–1.11	.121
		20–24	0.94	0.66–1.34	.729
Motivation for learning self-injection	Save money on transport	25+	Ref	—	—
		15–19	1.01	0.60–1.70	.971
		20–24	0.92	0.64–1.32	.656
	Convenience/saves time	25+	Ref	—	—
		15–19	0.56	0.34–0.93	.025
		20–4	0.83	0.57–1.20	.329
	Avoid future stockouts	25+	Ref	—	—
		15–19	1.24	0.68–2.28	.481
		20–24	1.03	0.69–1.53	.900
	Prefer to self-manage care	25+	Ref	—	—
		15–19	0.61	0.36–1.04	.070
		20–24	1.06	0.75–1.50	.758
	Discreet/can easily hide use	25+	Ref	—	—
		15–19	1.57	0.60–4.12	.359
		20–24	0.69	0.31–1.54	.361
	Learn a new skill	25+	Ref	—	—
		15–19	2.29	1.20–4.36	.012
		20–24	1.00	0.57–1.75	.996
	Provider recommended it	25+	Ref	—	—
		15–19	0.86	0.38–1.95	.718
		20–24	1.10	0.66–1.81	.723
	Partner recommended it	25+	Ref	—	—
		15–19	1.78	0.65–4.89	.262
		20–24	1.09	0.44–2.75	.849
	Friend(s) recommended it	25+	Ref	—	—
		15–19	3.64	1.22–10.84	.021
		20–24	1.78	0.61–5.18	.288

CI = confidence interval.

Rationale for learning to self-inject differed by age group. Although convenience and time savings were common motivations across all age groups, adolescents were significantly less likely to offer that rationale than adult women. Instead, adolescents were significantly more likely to report being motivated by wanting to learn a new skill and/or that friends had recommended SI (Table 2). Although not significantly more likely to mention enhanced discretion as a motivation for SI, a number of adolescents raised this topic in the IDIs.

*My thinking was that if I understand those things I can also self-inject without going to the facility*…. *I can go to a quiet place and inject without my parents knowing.* [18-year-old single female]

### Training format, preferences, and quality

About half of adolescents reported they were trained in a group, significantly more than older women, as expected given that the adolescent-focused program in Mubende District was implemented, in part, through the Safe Space platform (Table 3). About half of adolescents favored individual training, and more than one-third preferred group training. Adolescents were not significantly more likely than adult women to prefer individual training, despite their heightened concerns around privacy at clinics. Perspectives on group training focused largely on the value of shared experience.

*I trained with a group. It was a good experience because if you forget something the other person can remind you and says this is how they do this or that.* [18-year-old married female]

*I would love to be trained in a group because we would have collected together and asked at once, because when you are alone it may be hard to ask.”* [19-year-old married female]

**Table 3:** Training and counseling

	15–19 (n = 208)	20–24 (n = 302)	25+ (n = 550)
Training format			
Individual	34.6%	58.9%	60.7%
Group	50.5%	35.8%	34.5%
Both	14.9%	5.3%	4.7%
Preferred training format			
Individual	48.8%	57.6%	58.4%
Group	37.7%	25.5%	25.5%
No preference	13.5%	16.9%	16.2%
Training duration (mean, SD)	44.7 (33.2)	44.8 (27.9)	46.1 (30.4)
Type of provider conducting training			
Facility-based provider	59.1%	41.1%	30.2%
Village health team	40.9%	58.9%	69.8%
Method Information Index (during family planning counseling, client was told about…)			
All three MII factors as “Yes”	52.4%	62.6%	61.6%
Other family planning methods	77.9%	78.5%	76.9%
Risks/side effects from DMPA-SC	68.3%	83.4%	82.7%
What to do if experience side effects	75.5%	82.1%	82.0%

DMPA-SC = subcutaneous depot medroxyprogesterone acetate; MII = method information index; SD = standard deviation.

Those who preferred individual training often cited confidentiality and having more time to ask questions.

*[Self-injection training] requires to be alone because you catch quickly and at times you would want to talk about something private and fear to talk of it when you are many. You may share and then another person goes and says that so and so said this.* [18-year-old single female]

*Because some people have rumors, they go on telling different people and many people [in a training] may bring a problem to me, like, to a person who may want to marry me, when they hear such, they get to reconsider.* [14-year-old single female]

*The individual experience is better because the provider gives you time, whereby you can ask all that you need to know freely. When you are in a group setting, everyone is asking questions at the same time and questions cannot all be answered.* [19-year-old single female]

In all, 52.4% of adolescents received high-quality counseling as measured by the Method Information Index (Table 3); although adolescents were less likely to be told of side effects than older participants, these differences were not statistically significant. Method Information Index scores were similar for group (62.3%) and nongroup (59.2%) training (not shown).

### Comfort with SI

**Figure 1:**
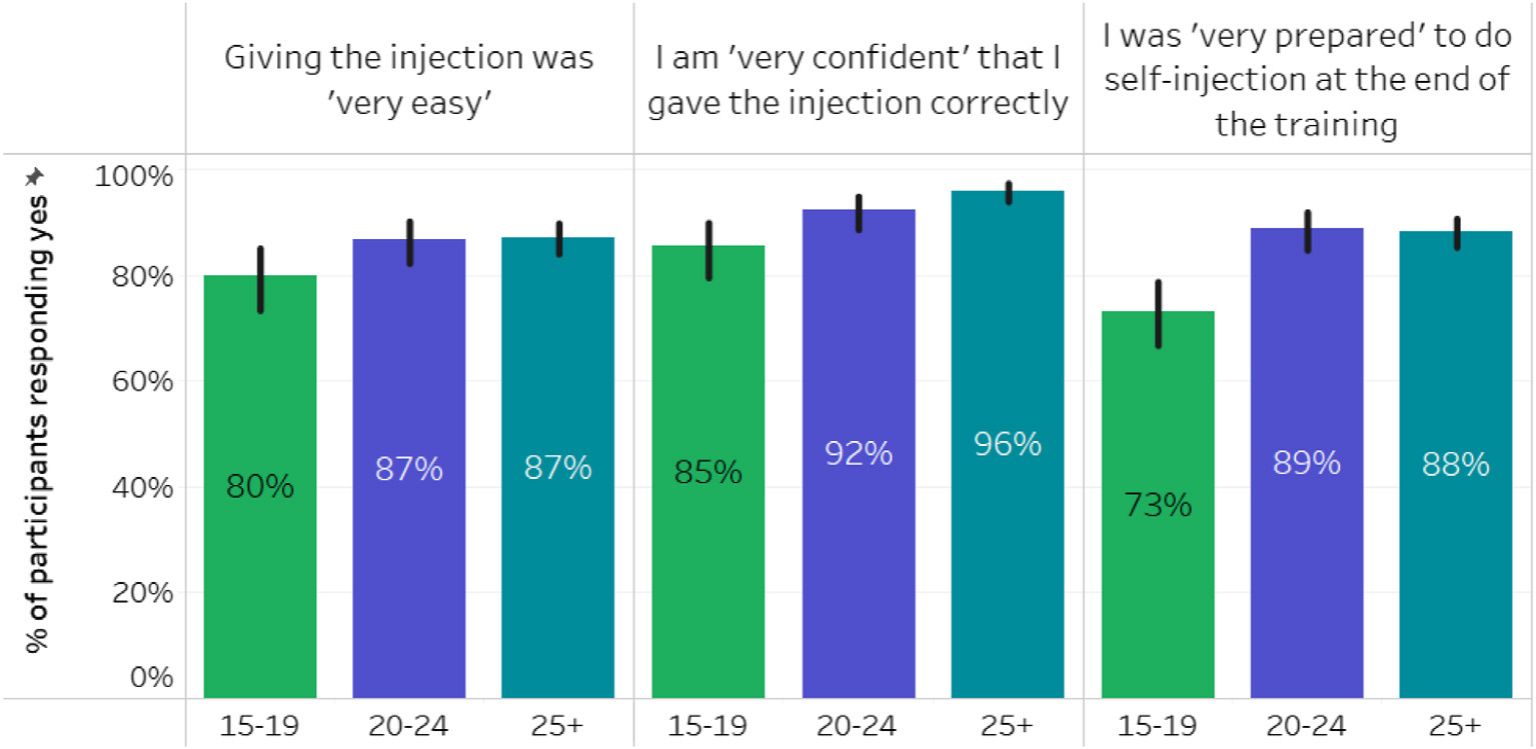
Readiness for self-injection posttraining.

Posttraining, adolescents were less likely to report that giving the injection was “very easy,” less likely to report high confidence they had injected correctly, and felt less prepared to self-inject independently (Figure 1). IDI comments suggest challenges with training quality.

*The truth is, I did not get a calendar, job aid, or puncture container. In the beginning, the provider was giving them out when I had just entered. I was trained after the tins had been given out. After the training I had not caught properly how to self inject. I told the provider to administer it, then the next one I will give myself.* [17-year-old single female]

*I learnt about it and was afraid at first, contemplating if I will be able to do it. I told myself in case I get another training I will be able to do it. After another trip to another Safe Space, I gained that. When I returned to the center to be trained again, I had courage and with practice on the condom modelmy courage was built the more.* [18-year-old single female] (The condom model is a condom filled with salt meant to be used as an anatomical model for the DMPA-SC injection site (such as thigh or abdomen)).

### Injection competence and continuation

Nearly 70% (69.7%) of all participants, including 60.9% of adolescents, demonstrated proficiency 4 months posttraining without additional guidance or instruction (not shown). In a multivariable mixed-effects logistic regression model controlling for education, adolescents were equally proficient at demonstrating the injection relative to older women (not shown). Although most were able to demonstrate injection proficiency, those who faced challenges mentioned difficulty with device activation (the most common error).

*I got the injection, held it like the provider had told me, opened it, mixed it, then after put it in my body, and it refused to put out the drug. I repeated but still it refused to put out the drug. So I left it.* [19-year-old married female]

Regarding continuation, 92.6% of all participants self-injected independently when due for reinjection (not shown). In a multivariable model that included education, marital status, and partner support, adolescents were not significantly more likely to discontinue either SI (Table 4, panel 2) or the injectable more generally (panel 1).

**Table 4:** Use of self-injection

Covariate	Receiving second dose of DMPA-SC (self-injection or provider administered)						Receiving second dose of DMPA-SC (self-injection only)					
	OR	95% CI	*p* value	aOR	95% CI	*p* value	OR	95% CI	*p* value	aOR	95% CI	*p* value
Age group												
25+	Ref	—	—	Ref	—	—	Ref	—	—	Ref	—	—
15–19	0.55	0.21–1.42	.214	0.54	0.20–1.44	.219	0.59	0.27–1.27	.177	0.53	0.23–1.18	.119
20–24	1.03	0.47–2.21	.950	1.14	0.51–2.56	.753	1.19	0.65–2.19	.579	1.21	0.63–2.33	.568
Married (yes)	1.65	0.83–3.30	.155	1.75	0.82–3.74	.149	1.73	0.97–3.11	.064	1.73	0.90–3.34	.099
Education (years)	1.02	0.92–1.13	.765	1.01	0.90–1.12	.903	1.06	0.97–1.16	.179	1.05	0.96–1.15	.317
Partner support for any family planning (yes, very)	1.28	0.69–2.38	.432	1.19	0.62–2.26	.603	1.60	0.95–2.67	.076	1.43	0.84–2.45	.187

n = 1,005 for both models.

aOR = adjusted odds ratio; CI = confidence interval; DMPA-SC = subcutaneous depot medroxyprogesterone acetate; OR = odds ratio.

Common myths surrounding injectable contraception may have particular impacts on adolescent continuation, given their low parity.

*I wasn’t able to re-inject because when I injected myself the first time, I got side effects and a certain health provider came to school and told us that Sayana Press injection has many side effects and it’s not good for young girls who have not given birth—that it burns their eggs. Then I said I won’t inject myself again. They advised that if am to use any family planning method I should use [intrauterine device] or implant.* [18-year-old single female]

## Discussion

The study did not find any significant differences in SI proficiency or continuation to a second injection between adolescents aged 15–19 years and adult women aged ≥25 years. The study found that compared with adult women, adolescents were more likely to be first-time modern contraceptive method users when they opted for SI. In terms of continuation, 86.1% of adolescents self-injected independently when due for their second injection.

These results may help fulfill Uganda's commitment to Family Planning 2030, which includes increased commitment and funding for adolescent reproductive health services. The government's 2012 Adolescent Health Policy Guidelines and Service Standards also address adolescents' access to SRH services following the principle, “Reproductive health services are a basic human right for all people including adolescents” [13]. These guidelines expired in 2015, and Uganda has faced challenges in implementing programs to improve adolescent SRH that address the lack of youth-friendly services at facilities, high travel costs and distance to services, lack of privacy, and persistent stigma or fear of discovery when obtaining services [7]. SI has the potential to address some of these challenges by providing a contraceptive option that adolescents can use competently, with fewer trips to health facilities.

Given adolescents in this study were able to self-inject at comparable levels to adult women, SI and other SRH self-care practices have the potential to contribute to reducing unmet need for contraception among this age group and improving well-being. We recommend adolescents be meaningfully incorporated into national SRH self-care strategies. This discussion section will highlight insights on contraceptive SI training and privacy preferences, continuation, and access.

### SI training and privacy preferences

Our results show that privacy remains a concern for adolescents. Adolescents expressed heightened concern over possible discovery while seeking contraceptives at a health center: 25.5% of adolescents reported being “very concerned” compared with 5.3% of adult women. Although the World Health Organization Global Standards for Quality Health-Care Services for Adolescents includes preventing staff from disclosing any adolescent information without consent [14], recent data from Uganda identifies privacy violation as the most significant concern about seeing a VHT [15].

Although respondents listed privacy as a concern, when asked about the motivation to self-inject, adolescents were not more likely to identify SI as more private. SI *can* increase privacy (by lessening contact with health facilities over time) but may not for young people, given the initial provider interaction and exposure risk during training. Moreover, SI may not be discretion enhancing if there is no privacy at home nor a secure place to store units.

Despite heightened concerns around lack of privacy at clinics, adolescents were not significantly more likely than adult women to prefer individualized over group training. They varied in their training location preferences. Burke et al. make the case through qualitative inquiry that adolescents prefer individual over group training; however, our quantitative results did not reveal significant differences in their preferences [16]. Indeed, a sizable share desired group training.

This suggests there is no one-size-fits-all adolescent services approach. Given that services offered in adolescent spaces are difficult to sustain and scale [5], it is critical to organize health services through a variety of formats to fit diverse client preferences. Importantly, health services managers can ensure that groups are age sensitive (e.g., adolescents are not mixed with older women), and when providers offer group training, they should provide individual time with clients afterward.

Given that following SI training, adolescents felt less prepared to self-inject independently and were less likely to report that giving the injection was “very easy,” family planning programs newly offering SI should include specific provider training content on SI counseling that is developmentally appropriate for adolescents. Adolescents often have less knowledge about sexuality and contraception than adults and have less experience engaging in aspects of self-care. Therefore, different and more robust approaches to build confidence in their new skills are needed as part of SI training. Furthermore, we recommend that messaging around DMPA-SC SI be differentiated for adolescents highlighting the benefits of learning a new skill, as this was a more pronounced interest among adolescents interested in learning to self-inject.

### Continuation

Our study found that continuation of DMPA-SC (self-injected or not) among 15- to 24-year-olds was not significantly different from that of older women. This varies from other literature on contraceptive continuation that suggests adolescents are more likely to discontinue use [17] but is consistent with recent research suggesting SI may increase continuation for young women. In a 2018 study in Uganda, continuation rates for young self-injectors (18–24 years) were indistinguishable from adult self-injectors and significantly greater than continuation rates for young women receiving DMPA from a provider [18], suggesting that SI conveyed a particular advantage for youth. Our results offer an additional perspective from a younger cohort, beginning at age 15 years. A 2020 study in Malawi also found no significant difference in continuation by age (18–24 years compared to ≥25 years) among self-injectors at 12 months [12].

Although our findings importantly indicate that SI is an acceptable contraceptive option for young people's use in relation to adult women, it is limited given the study did not capture additional critical details on youth's contraceptive intentions and experiences of informed choice. Researchers should bear in mind that adolescent sexual relationships can be more transitory, and unmet need for contraception may fluctuate at this life stage. The study results do not account for discontinuation “while still in need,” an important measure for youth, as for all women. We must continue to refine indicators for contraceptive continuation “that make autonomy and respect for rights the primary outcome of interest” [19].

Our study is further limited for following adolescents for only one follow-up injection. More research is needed to explore when and why adolescents discontinue use and to evaluate the extent to which SI may be particularly advantageous for adolescent continuation.

### Access to SI

Adolescents were significantly (27%) less likely than adult women to report first hearing about SI from a VHT. Conversely, the odds of adolescents hearing about SI from friends or family were about twice as likely as for adult women.

Since VHTs began offering family planning in 2011, little reporting has surfaced on how well they meet the family planning needs of young people [15]. Our finding that adolescents and youth 15–24 years were less likely to report they first heard about SI from a VHT suggests either that community health workers are less likely to serve young clients or they withhold SI as an option during their counseling sessions with younger women. The latter would constitute provider-imposed restrictions on adolescent access to SI, supporting previous studies that documented providers' hesitancy to offer this option for young people. Cover et al. describe providers' expectations that young women who self-inject will engage in premarital sex or promiscuity, misconceptions that injectables cause infertility, and reservations about offering SI to unmarried adolescents without parental consent (although no policy requiring parental consent exists). A common reason for opposition to adolescent SI: adolescents do not have the maturity to manage it [20]. However, measuring provider bias is highly challenging [21], constituting a limitation to our study methodology.

The World Health Organization's 2015 Medical Eligibility Criteria for Contraceptive Use states: “Age alone does not constitute a medical reason for denying any method to adolescents” [22]. Age-based discrimination is also considered a barrier to self-care practices [3]. Providers can create barriers to choice, typically based on client characteristics or a contraceptive method, the most common bias is against the provision of full method choice to youth [21,23]. Hormonal methods including injectables have been noted to face method-specific biases [23]. The denial of adolescent sexuality is well documented as a persistent global challenge, leading adolescents in many contexts to learn about SRH from peers [2], consistent with our study. Plesons et al. write, “refusal often manifests as disrespect and judgment from health service providers when adolescents seek services, as well as powerful—often paralyzing—opposition to [adolescent] [sexual and reproductive health and rights] policies, programs, and services” [2]. Thus, it remains apparent that pertinacious social and cultural sentiments around adolescent sexuality and contraceptive use need to be negotiated.

Given the vast potential of community health workers to offer DMPA-SC and SI, as well as other self-administered methods, we recommend adolescents be increasingly more included in community health worker family planning efforts. Additional resources and routine measurement practices are needed to support community health workers to reach adolescents. Where there are no legal restrictions on the provision of contraceptives related to age or marital status, health workers must be informed of this and of their obligation to respect these laws so that young people are ensured unrestricted access.

Study limitations include participants aged 15–17 years were only included if they were emancipated minors, limiting the generalizability of our results to nonemancipated minors in this age group. Emancipated minors made up less than 20% of the 15- to 19-year-old study population. Continuation results only reflect the follow-up of clients through one reinjection period. Although we see some indication of provider bias among VHTs, our study design was not ideal for measuring the extent of bias, a simulated client survey would be a more effective approach.

### Conclusion

This study indicates that contraceptive SI is a promising method of contraception for adolescents in Uganda, given comparable proficiency and continuation relative to adult women. Adolescents should be actively included in accessing self-care for SRH and rights, and health policies and program managers should ensure rights-based access to a range of methods, including SI for this age group.
